# Cryptococcal infection presenting as soft tissue abscess and arthritis: Case report

**DOI:** 10.1097/MD.0000000000026656

**Published:** 2021-07-16

**Authors:** Yong Jin Cho, Song Iy Han, Sung-Chul Lim

**Affiliations:** a Department of Orthopedic Surgery; b Division of Premedical Science; c Department of Pathology, College of Medicine, Chosun University, Gwangju, Korea.

**Keywords:** abscess, arthritis, cryptococcosis, pseudogout

## Abstract

**Rationale:**

Cryptococcal infection has been documented in immunocompromised patients. AIDS and renal transplant recipients account for majority of the cases. Most cases present with central nervous system or disseminated disease, with only few presenting soft tissue, bone, and joint manifestations.

**Patient concerns:**

We present a case of soft tissue mass in a 66-year-old female renal transplant recipient and that of arthritis in a 64-year-old immunocompetent man who presented pseudogout arthropathy. Chest radiographies of both cases were negative. Biopsy revealed cryptococcal organisms. Blood culture or cerebrospinal fluid sampling indicated positive results for cryptococcal antigen.

**Diagnosis:**

*Cryptococcus neoformans* was recovered in the wound culture.

**Interventions:**

The patients received intravenous fluconazole and flucytosine, followed by oral fluconazole administration.

**Outcomes:**

Symptomatic improvements were achieved and no subsequent relapses were observed.

**Lessons:**

The authors experienced 2 cases of cryptococcosis with very unusual clinical presentation. Early clinical suspicion and serum cryptococcal antigen testing can help in rapid appropriate diagnosis in immunocompetent as well as immunocompromised patients even in the absence of pulmonary involvement.

## 1 Introduction

Cryptococcosis is an infectious disease caused by *Cryptococcus neoformans* or *Cryptococcus gattii,* which are encapsulated yeast-like fungus and are widely distributed in the soil, birds dropping, and spoiled milk. As the fungus generally enters the body through inhalation, it invades lungs and spreads to the central nervous system (CNS) or other organs via lymphatic or blood vessels. In general, infection in immunodeficient patients occurs through *C. neoformans*; however, infections from *C. gattii* are more common for immunocompetent patients who reside at temperate zones. The majority of disseminated cryptococcal infections occur in patients with acquired immune deficiency syndrome (AIDS); however, 11% to 14% infection occur in patients without AIDS. Moreover, majority (75%) of non-AIDS related cases occur in patients with other immune deficiencies.^[1–4]^

If a clinical manifestation of cryptococcosis is observed as soft tissue mass, bone destruction (osteomyelitis), or arthritis, which are remarkably rare, there may be considerable delays in accurate diagnosis and appropriate treatment. Furthermore, the manifestations may be mistaken for malignant tumors, which may lead to unnecessary surgery.

In general, 1 to 3 cases of isolated skeletal cryptococcosis have been continuously reported in the literature,^[5]^ and reports of skeletal cryptococcosis with associated soft tissue abscess have also been published^[1]^; however, the cases with soft tissue mass without skeletal lesion are rare.

In 1 case, cryptococcosis was confirmed via computed tomography (CT)-guided core biopsy in a patient who was clinically suspected to have malignant tumor due to pathologic fracture of the rib and mass in the surrounding soft tissue.^[6]^ In another case, left patellar osteomyelitis and intramuscular abscess involving distal left quadriceps was diagnosed as sepsis and initially treated with broad-spectrum antibiotics.^[7]^ The broad-spectrum antibiotics did not improve the symptoms, and the patient was diagnosed with cryptococcosis 46 days after the onset of symptoms. Thereafter, the patient was administered antifungal therapy, but he died. Both the aforementioned cases involved immunocompromised patients; however, disseminated cryptococcal infections have also been reported in immunocompetent hosts. In 1 case, cryptococcosis was confirmed via CT-guided core biopsy in a patient with osteomyelitis of the mandible, manubrium, and third rib with associated soft tissue abscesses.^[1]^

Concurrent gout and cryptococcal arthritis have been reported in renal transplant patients,^[8]^ and arthritis involving hip, elbow, knee, or sternoclavicular joints have also been reported in immunocompromised conditions.^[9–12]^ Moreover, cryptococcal arthritis has also been reported in an immunocompetent 24-year-old male without a defined medical history.^[13]^

Herein, the authors experienced 2 cases of cryptococcosis with very unusual clinical presentation. Cryptococcosis was diagnosed in soft tissue mass from a renal transplant recipient and cryptococcal arthritis was diagnosed in an immunocompetent patient who was being treated with concurrent pseudogout and arthritis, respectively. Prompt and accurate diagnosis not only avoids unnecessary surgery but also determines the success or failure of the treatment. Therefore, a review of literature was conducted to examine the clues in order to enable accurate diagnosis based on the various clinicopathological findings observed in the cases presented in this study.

## 2 Case report

This study was approved by the institutional review board of the Chosun University Hospital (Permission number: CHOSUN 2021-01-019). Informed written consents were obtained from the patients for publication of this case report and accompanying images.

### 2.1 Case 1

A 66-year-old woman was presented to the hospital with a subcutaneous soft tissue mass on her left thigh as the chief complaint. She was undergoing an immunosuppressive regimen as a renal transplant recipient, and was being administered with medications for type II diabetes mellitus and hypertension. No fever was noted at the time of presentation, and no abnormal findings were recorded other than anemia with hemoglobin of 10.3 g/dL, hematocrit of 32.8%, and red blood cells of 3.23 × 10^6^/μL. White blood cell (WBC) count was 8.76 x 10^3^/μL. Differential cell counts were neutrophil 68.8%, lymphocyte 3.4%, monocyte 9.7%, eosinophil 6.9%, basophil 0.5%, and large unstaind cell (LUC) 0.8%. Serologic tests including cytomegalovirus (CMV), venereal disease research laboratory (VDRL), hepatitis B surface antigen (HB_s_Ag), hepatitis B surface antibody (HB_s_Ab), human immunodeficiency virus antigen (HIV-Ag), and human immunodeficiency virus antibody (HIV-Ab) were negative.

**Figure 1:**
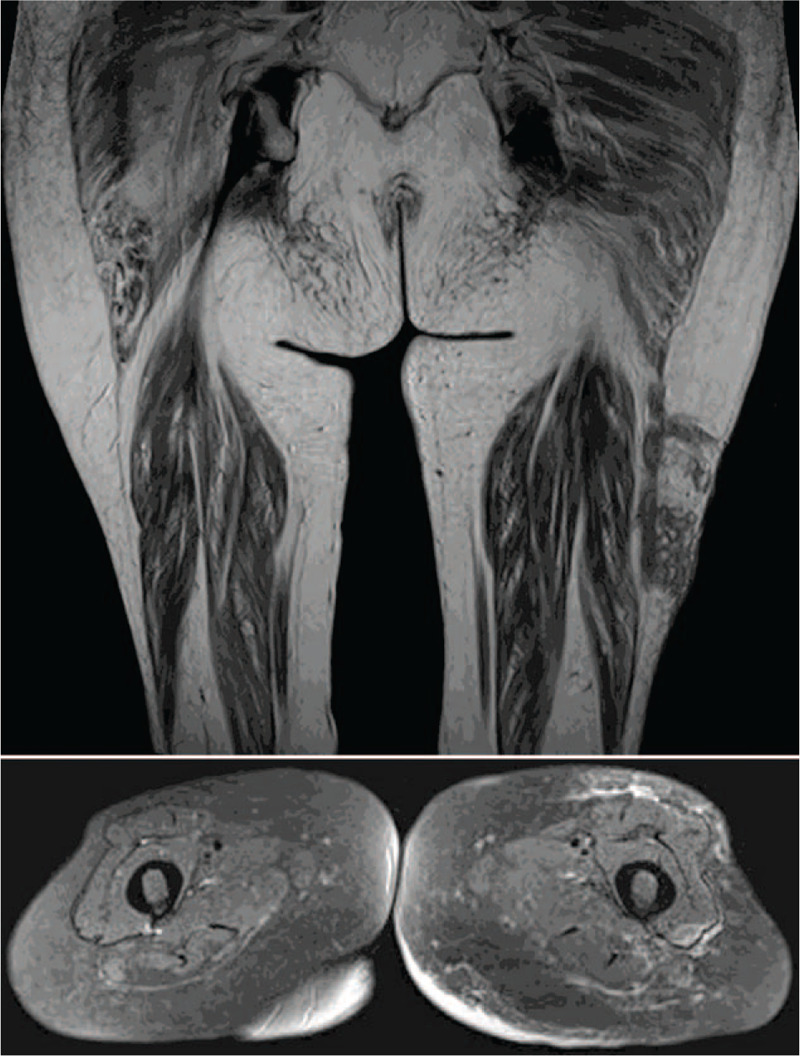
Case 1. MRI showed a lobular margined 15 cm mass in the intermuscular septum between left vastus lateralis and biceps muscles.

Magnetic resonance image (MRI) revealed lobular 15 cm mass in the intermuscular septum between left vastus lateralis and biceps muscles (Fig. 1). Chest radiography was negative. Clinical impression indicated hemangioma, nodular fasciitis, or other soft tissue tumor.

**Figure 2:**
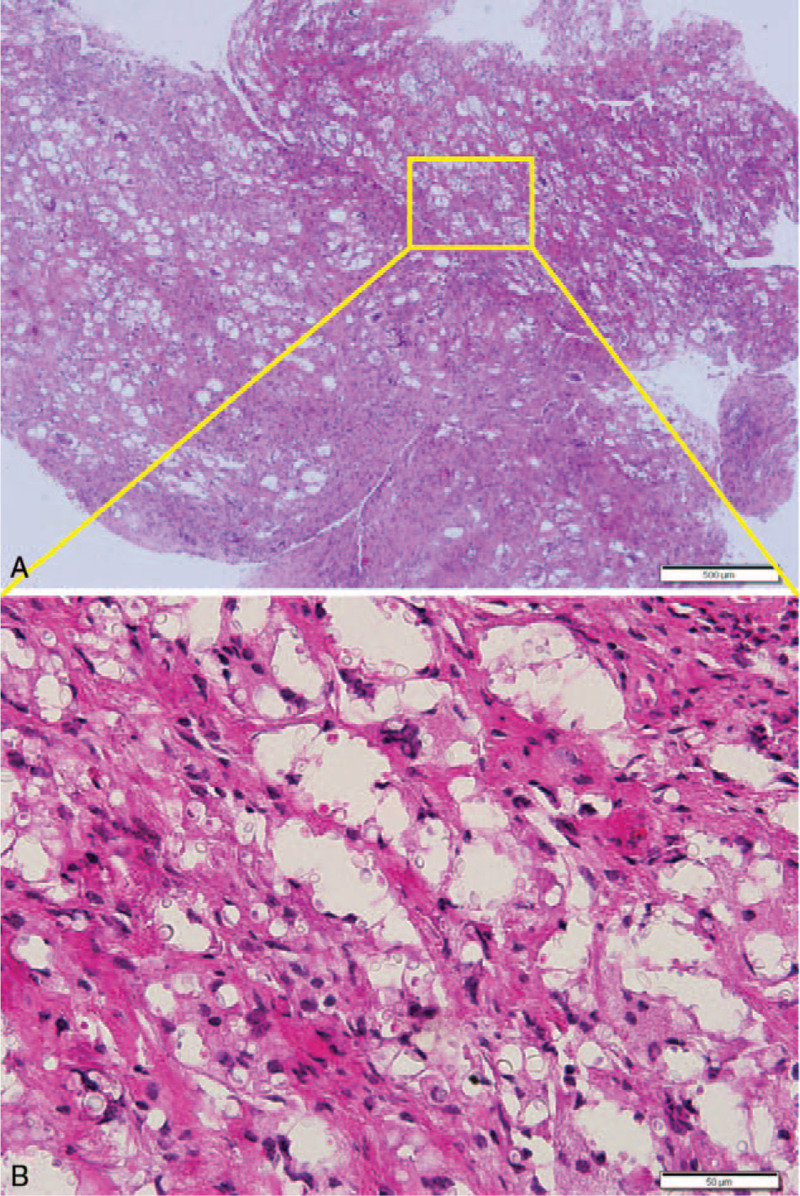
Histopathological findings of the Case 1. (A) Low-power image showed chronic granulomatous inflammation consisted of microcystic spaces, lymphohistiocytes and multinucleated giant cells. (B) High-power view of the area in A showed a lot of spherical shaped capsulated structures in the microcystic spaces. Scale bars measure 500 μm (A) and 50 μm (B).

**Figure 3:**
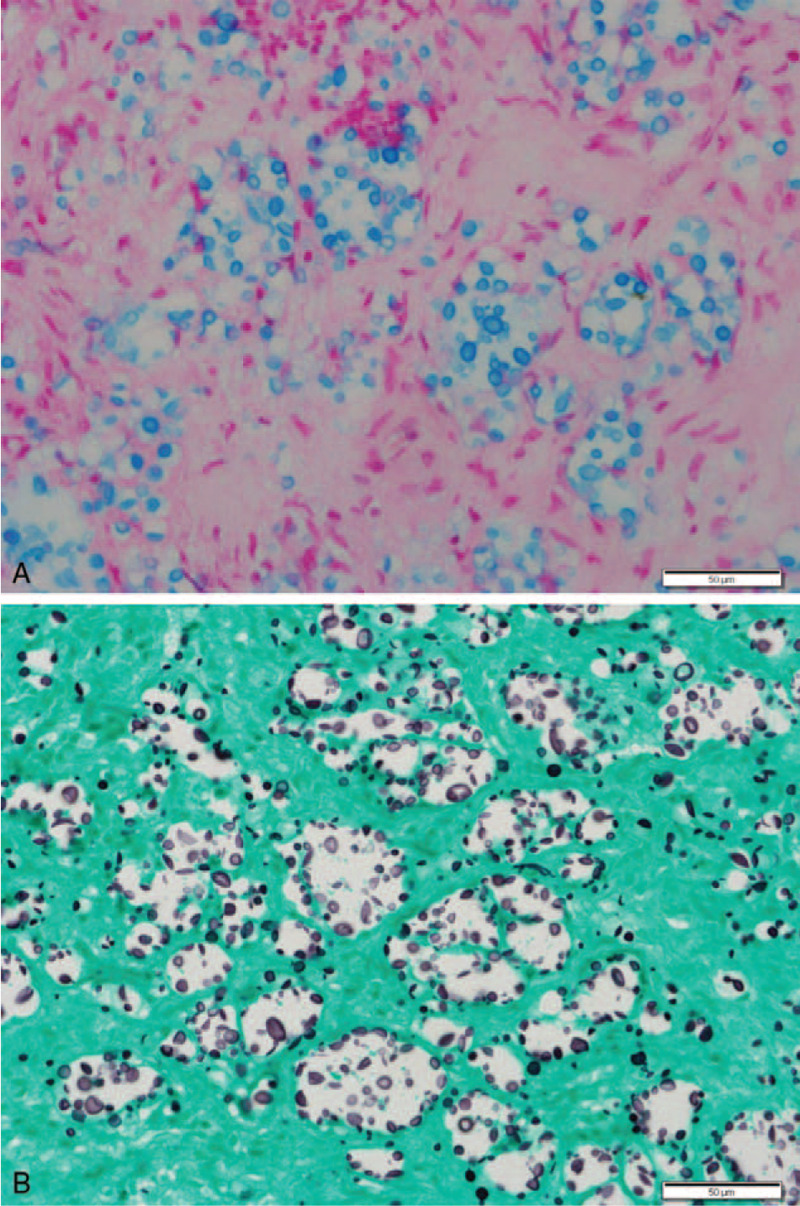
Special stainings of the Case 1. (A) Alcian blue staining highlighted encapsulated yeasts. (B) GMS staining showed yeast forms with narrow-based budding. Scale bars measure 50 μm.

This case was an unusual clinical presentation, which was presumed to be a neoplastic condition rather than an infectious disease. Therefore, the final diagnosis was delayed due to various workups considering surgical removal, and excisional biopsy of the lesion was performed. On histopathologic examination, the entire resected mass was revealed to be chronic granulomatous inflammation accompanied by fibrosis; however, the mass was not a well-formed granuloma observed in tuberculosis or sarcoidosis, but microcysts of various sizes were diffusely arranged under fibrosis background. Multinucleated giant cells appeared to be scattered in the microcysts, and therefore, the formation of vague granuloma in the surrounding was barely recognizable. When high-power microscopic examination was performed to determine the identity of granuloma, numerous spherical structures of various sizes smaller, similar, or larger compared with the size of a lymphocyte were observed, and they appeared to be slightly refractile (Fig. 2). They were diagnosed as *Cryptococcus* because the capsules were strongly positive to periodic acid Schiff (PAS), mucicarmine, and Gomori methenamine-silver stains (Fig. 3). Blood culture and cerebrospinal fluid (CSF) sampling revealed positive results for cryptococcal antigen. *C. neoformans* was recovered in the wound culture. Detailed neurologic examination for CNS revealed no meaningful neurologic sign and symptoms.

She was treated with intravenous fluconazole (600 mg/day) and flucytosine (1500 mg every 6 hours) for 2 weeks, followed by oral fluconazole (400 mg/day) administration for six months. One year after discharge, the patient was doing well with complete resolution of symptoms.

### 2.2 Case 2

**Figure 4:**
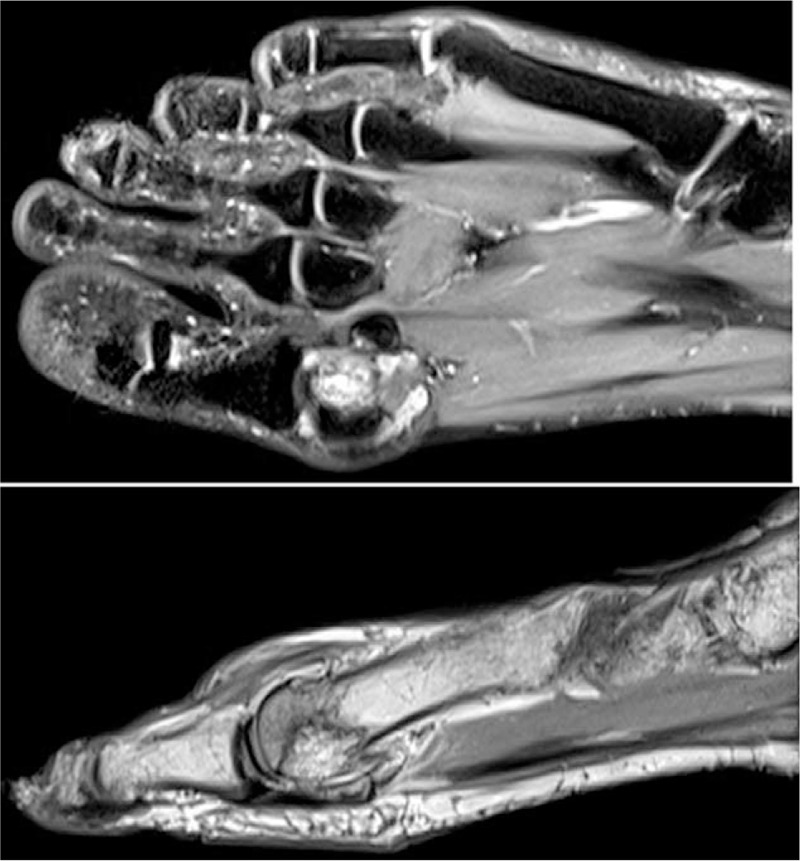
Case 2. MRI showed irregular infiltrating soft tissue mass at around of left first metatarsal-phalangeal joint causing bony destruction/erosion and osseous involvement.

A 64-year-old male patient was presented to the hospital with swelling accompanied by pain in the left big toe as the chief complaint. There were no specific findings in the patient's history other than the pheochromocytoma surgery of the right adrenal gland performed 20 years ago and the pulmonary emphysema diagnosed one year ago. No fever was recorded during presentation, and the findings of peripheral blood were as follows; erythrocyte sedimentation rate (ESR) 20 mm/h, hemoglobin 16.1 g/dL, hematocrit 49.3%, and red blood cells 5.16 × 10^6^/μL. WBC count was 11.28 x 10^3^/μL. Differential cell counts were neutrophil 59.2%, lymphocyte 27.4%, monocyte 12.2%, eosinophil 0.9%, and basophil 0.3%. Serologic tests for VDRL, CMV, HB_s_Ag, HB_s_Ab, and HIV were negative. The findings on MRI revealed abnormal signal intensity, enhancing irregular infiltrating soft tissue mass around the left first metatarsal–phalangeal joint causing bony destruction/erosion and osseous involvement (Fig. 4). It was clinically considered as soft tissue sarcoma, and depositional/biomechanical diseases such as gout were also considered necessary as differential diagnosis. Pain was noticed in the big toe approximately 3 years and 6 months ago. The uric acid in blood at that time was 7.8 mg/dL, which indicated that this pain was due to acute gout attack. This was followed by medication treatment. Drug treatment was continued for gout due to recurrent pain in the ankle and foot. This condition was diagnosed as pseudogout 1 year ago via polarizing microscopic examination of the joint fluid in the pain area. The uric acid in blood at that time was normal at 4 mg/dL.

**Figure 5:**
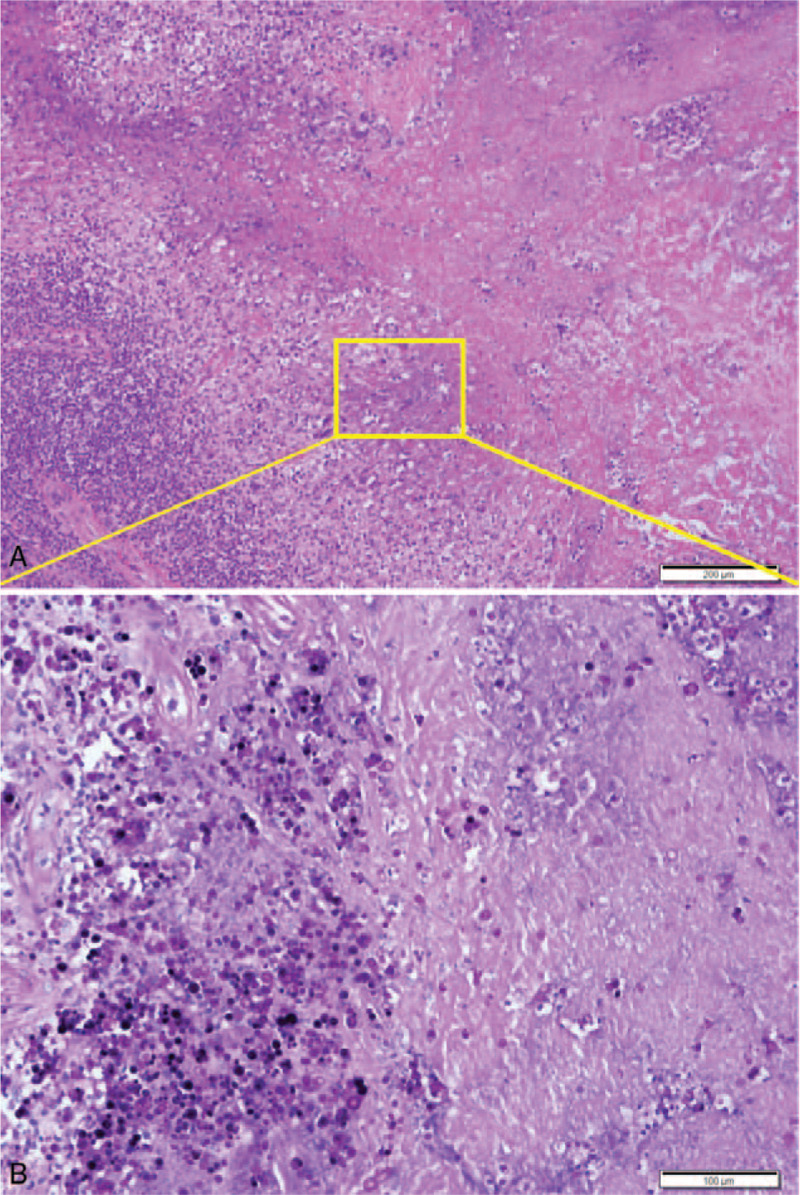
Histopathological findings of the Case 2. (A) Low-power image showed extensive caseous-like necrosis with vague palisading chronic granulomatous inflammation consisted of lymphohistiocytes, foam cells and multinucleated giant cells. (B) High-power view of the area in A showed variable sized a lot of spherical shaped structures in and around the necroses. (A) Hematoxylin and eosin stain, (B) PAS stain. Scale bars measure 200 μm (A) and 100 μm (B).

Resection of the left big toe lesion was performed. Majority of the lesions exhibited severe necrosis, and living tissue was observed at the edges. The necrosis was found to be similar to caseous necrosis. Severe lymphohistiocyte infiltration was observed in the surrounding living tissues, and there was formation of vague granulomatous inflammation with foam cells and multinucleated giant cells. In the cytoplasm of foam cells and multinucleated giant cells, spherical structures with clear or unclear outlines were noticed (Fig. 5A). The nested-polymerase chain reaction (PCR) test for *Mycobacterium tuberculosis* presented negative findings, which ruled out tuberculosis. The spherical structures were observed in various sizes under high-power microscope, and were diagnosed as *Cryptococcus* because the capsule was found to be strongly positive for PAS, mucicarmine, and Gomori methenamine-silver stain (Fig. 5B). Blood culture results were positive for cryptococcal antigen. *C. neoformans* was recovered in the wound culture. No gout or pseudogout was found on histopathologic findings. Therefore, whether the cause of pain, which started 3 years and 6 months ago, was gout or pseudogout remains unclear, and the possibility that cryptococcosis continued and progressed from 3 years and 6 months ago could not be dismissed. He was treated with intravenous fluconazole (600 mg/day) and flucytosine (1500 mg every 6 hours) for 2 weeks, followed by oral fluconazole (200 mg/day) administration for 6 months with progressive clinical improvement. One year after discharge, bone scan revealed no active osteomyelitis or arthritis.

## 3 Discussion

*Cryptococcus* is a ubiquitous yeast that can cause disease in healthy hosts; however, the infection mostly occurs in patients with immunodeficiencies; advanced AIDS patients are most commonly infected by this yeast.^[1–4]^

When unexpected clinical manifestation appears with cryptococcosis, delayed diagnosis leading to missed treatment periods or performances of unnecessary surgical procedures may occur. Even if a histopathologic examination is conducted, it is difficult to diagnose this disease or the results may be mistaken for another disease. A case of spindle cell lesion admixed with inflammatory cells accompanied with bone destruction was observed, which led to clinical and pathological suspicion of being malignant tumors, and this required differential diagnosis such as inflammatory leiomyosarcoma, inflammatory myofibroblastic tumor, and lymphoma.^[6,14]^ In this case, multiple immunohistochemical stains were performed for differential diagnosis. The spindle cell that comprises the lesion was positive for CD68, which led to workup focusing on the potential infectious etiology.

In Case 1, there was fibrosis but no cellular atypia, and the occasional multinucleated giant cells were suspected to present an infectious condition than a neoplastic condition. In Case 2, necrosis was severe, and foam cells and multinucleated giant cells were arranged in a pseudopalisading pattern along the necrotic margin, which also led to suspicion of an infectious condition. Both cases were fundamentally presumed to be chronic granulomatous inflammation; however, granuloma was indiscernible except through the scattered multinucleated giant cells. Nevertheless, both cases revealed spherical structures of various sizes in the cytoplasm of multinucleated giant cells or foam cells, and around the multinucleated giant cells. Therefore, PAS, mucicarmine, and Gomori methenamine-silver stains majorly helped in identifying the structure during diagnosis.

When cryptococcosis manifested in the form of a soft tissue mass, it was mainly accompanied by invasion of the surrounding bone. Although the temporal relationship cannot be accurately determined, it is presumed that osteomyelitis preceded and extended to the surrounding soft tissue with or without pathological fractures to produce a soft tissue mass in the form of a cryptococcal abscess^[1,6,7]^; however, the presented Case 1 is an extremely rare case as the manifestation only occurred as intermuscular mass without invasion from a surrounding bone.

Cryptococcal skeletal lesion occurs in 5% to 10% of disseminated cases, often involving bony prominences. The most common sites are vertebrae, femur, tibia, and rib, in the given order,^[1]^ and moreover, arthritis or osteomyelitis are rarely incurred alone.^[1,15,16]^

As isolated skeletal cryptococcosis has been reported in immunocompromised patients as well as in immunocompetent hosts,^[5]^ it is crucial to include cryptococcosis in differential diagnosis, even in cases with unusual clinical manifestations. Furthermore, it is difficult to determine whether the presented Case 2 reveals cryptococcosis complicated by gout or pseudogout arthrosis; however, concurrent gout and cryptococcal arthritis have been reported in a renal transplant.^[8]^ Therefore, if symptoms do not improve or the disease progresses despite administering medications for gout, it will be necessary to check the possibility of cryptococcosis.

The standard treatment is not yet established but it is recommended to start with aggressive intravenous antifungal injection and continue with a suppressive treatment orally during a variable time depend on the patient status. Surgical indication is considered in lesions that affect the spinal stability, deformity or neurological compromise, degenerative symptomatic arthritis, and some cases of meningitis, and for local infectious control.^[17]^ Many authors have recommended systemic chemoprophylactic treatment preoperatively and postoperatively for patients who have had fungal arthritis, and it may be argued that antifungal chemotherapy should be used at the time of joint surgery in a patient who has had cryptococcal arthritis, even if the patient has been apparently free of the disease for a long period of time.^[9]^

The usual prognosis with treatment is good, except in those patients with other concomitant disseminated opportunistic infections.^[10]^

Cryptococcosis is frequently reported in patients with sarcoidosis,^[5,12]^ which occurs as an opportunistic infection due to administration of immunosuppressive drugs such as steroids, methotrexate, azathioprine, and cyclophosphamide and tumor necrosis factor alpha inhibitors.^[18,19]^ In addition, sarcoidosis itself is considered to be an independent risk factor for cryptococcal infections.^[20]^ Sarcoidosis forms noncaseating granulomas; however, diagnosis of cryptococcosis may be overlooked if it concurrently occurs. Therefore, efforts should be made to identify *Cryptococcus* in sarcoidosis-like lesions by recognizing the relationship between sarcoidosis and cryptococcosis.

## 4 Conclusion

If cryptococcosis clinically manifests as soft tissue mass, bone destruction, or arthritis, which is extremely rare, considerable delays may occur in accurate diagnosis and appropriate treatment, or the manifestations may be mistaken for malignant tumors, thereby leading to unnecessary surgery. If vague granuloma with multinucleated giant cells is observed in immunocompetent hosts in addition to immunocompromised patients, it will be necessary to closely observe whether spherical structures of various sizes are present in the cytoplasm of multinucleated giant cells or foam cells, or around multinucleated giant cells. PAS, mucicarmine, and Gomori methenamine-silver stains can be used to diagnose this structure, and if a neoplastic condition is suspected due to severe spindle cell proliferation, positive immunohistochemical staining for CD68 is considered as a useful to mark the potential increase in the infectious condition.

## Author contributions

**Data curation:** Yong Jin Cho, Song Iy Han.

**Funding acquisition:** Sung-Chul Lim.

**Methodology:** Yong Jin Cho, Song Iy Han, Sung-Chul Lim.

**Supervision:** Sung-Chul Lim.

**Validation:** Sung-Chul Lim.

**Writing – original draft:** Yong Jin Cho.

**Writing – review & editing:** Song Iy Han, Sung-Chul Lim.
